# Skill Differences in a Discrete Motor Task Emerging From the Environmental Perception Phase

**DOI:** 10.3389/fpsyg.2021.697914

**Published:** 2021-10-01

**Authors:** Yumiko Hasegawa, Ayako Okada, Keisuke Fujii

**Affiliations:** ^1^Faculty of Humanities and Social Sciences, Iwate University, Morioka, Japan; ^2^Japan Ladies Professional Golfers’ Association, Tokyo, Japan; ^3^Graduate School of Informatics, Nagoya University, Nagoya, Japan; ^4^RIKEN Center for Advanced Intelligence Project, RIKEN, Fukuoka, Japan; ^5^PRESTO, Japan Science and Technology Agency, Kawaguchi, Japan

**Keywords:** slope perception, decision-making, alignment error, kinematics, golf putting

## Abstract

Because of the challenges associated with measuring human perception and strategy, the process of human performance from perception to motion to results is not fully understood. Therefore, this study clarifies the phase at which errors occur and how differences in skill level manifest in a motor task requiring an accurate environmental perception and fine movement control. We assigned a golf putting task and comprehensively examined various errors committed in five phases of execution. Twelve tour professionals and twelve intermediate amateur golfers performed the putting task on two surface conditions: flat and a 0.4-degree incline. The participants were instructed to describe the topographical characteristics of the green before starting the trials on each surface (environmental perception phase). Before each attempt, the participants used the reflective markers to indicate their aim point from which the ball would be launched (decision-making phase). We measured the clubface angle and impact velocity to highlight the pre-motion and motion errors (pre-motion and motion phase). In addition, mistakes in the final ball position were analyzed as result errors (post-performance phase). Our results showed that more than half of the amateurs committed visual–somatosensory errors in the perception phase. Moreover, their aiming angles in the decision-making phase differed significantly from the professionals, with no significant differences between slope conditions. In addition, alignment errors, as reported in previous studies, occurred in the pre-motion phase regardless of skill level (i.e., increased in the 0.4-degree condition). In the motion phase, the intermediate-level amateurs could not adjust their clubhead velocity control to the appropriate level, and the clubhead velocity and clubface angle control were less reproducible than those of the professionals. To understand the amateur result errors in those who misperceived the slopes, we checked the individual results focusing on the final ball position. We found that most of these participants had poor performance, especially in the 0.4-degree condition. Our results suggest that the amateurs’ pre-motion and strategy errors depended on their visual–somatosensory errors.

## Introduction

Skill science research revealed that individuals with excellent skills have higher performance accuracy and reproducibility. They also have an abundance of task-specific knowledge and a cognitive function that underpins their skillful movements, enabling them to rank tactics and select them quickly (e.g., de Groot, 1965; Williams et al., 1999, 2003; Lex et al., 2015). However, due to the difficulty in measuring human perception and strategy, studies have not thoroughly explained the process from perception to motion to performance results. That is, scholars have yet to clarify whether unskilled people cannot perceive their environment well, whether they cannot choose good strategies, or whether they do not merely acquire appropriate movements to play well. To advance the motor skills of unskilled individuals, researchers and coaches need to understand the bottlenecks impeding their progress. We believe it is critical to comprehensively investigate the errors that occur in each phase of one skill.

There are several models of human error (Tous-Ral and Liutsko, 2014), and Schmidt’s model (Schmidt et al., 2019) has been used often in motor learning research. That is, a renowned classical framework is Schmidt’s information-processing model, which consists of the stimulus (input), stimulus identification, response selection, response programming, and movement (output) (Schmidt et al., 2019). These computer metaphors led to many notable human movement science studies that examine the perceptual-cognitive process and movement. In particular, there has been an increase in studies that capture perceptual-cognitive expertise in sports, using methods such as eye movement recording, film occlusion, and point-light displays, and verbal protocol analysis to identify its underlying mechanism (Williams and Ericsson, 2005).

Meanwhile, the appropriateness and resolution of the performer’s perception are not understood fully due to a lack of relevant research in sports. Scholars have suggested that human perception changes dynamically. For example, even in the same environment, individuals perceive the size and height of a target differently depending on their situation (e.g., Witt and Proffitt, 2008; Witt et al., 2008; Lee et al., 2012) and psychological state (Stefanucci and Proffitt, 2009). On the other hand, motor learning is described as a lasting improvement in performance compared to a baseline measure that can be attributed to training (Shmuelof et al., 2012; Sigrist et al., 2013; Bienkiewicz et al., 2019). Motor learning has been associated with systematic changes in proprioception (Haith et al., 2008; Ostry et al., 2010; Wong et al., 2012) and generates accurate movements, improving sensory acuity (Wong et al., 2011). Specifically, proprioception enable them automatically after many repetitions (skill acquisition). This means less control of attention and cognition, which results in higher velocity (Jordan, 1972; Schmidt et al., 2019). These studies suggest that motor learning may allow a person’s perceptual system to respond more accurately and sensitively to their movement and the environment. The difficulty in measuring performer subjectivity has led to few studies but remains an issue that researchers must address. Golf putting is an appropriate task for investigating such problems. It is a discrete motor skill in which phases can be classified, that is, from environmental perception to the end of skill execution. Based on Schmidt’s information-processing model, we investigated the golf putting motor task to classify its five phases—the perceptual phase of the environment, the decision-making phase, the pre-motion phase, the motion phase, and the post-performance phase. We comprehensively examined the errors that occurred in each one.

Successful putting entails more than proficient movement control. It requires, among other factors, the ability to perceive slight differences in the environment (van Lier et al., 2011). Karlsen and Nilsson (2008) described the factors that influence putting direction in the chronological order of green reading (60%), aiming, stroke (34%), and green inconsistencies (6%). Green reading is the process of finding the correct initial ball direction by evaluating the green’s surface and topographical characteristics (Karlsen et al., 2008). Simply put, green reading represents the combination of the surface and topographical perception phase and the decision-making phase. Empirically, golf instructors often feel that unskilled people cannot perceive green surfaces and topography. Thus, we experimentally set slightly different slope conditions and assessed whether the performer can perceive them. Previous research determined a performer’s size and height perception by manipulating a miniature model (Witt and Dorsch, 2009), drawing life-sized replicas of the target (Wood et al., 2013), or selecting one of several miniature figures (Lee et al., 2012). In this study, we asked participants to freely move around the green as they would in normal play and verbally express their slope perception (Campbell and Moran, 2014). Thus, an error during the environmental perception phase would be a visual–somatosensory error.

Following the perception phase, the main issue in the decision-making phase is deciding how much force to use and which direction to launch the ball. A study that investigated gaze on sideward-slope putting reported that increasing the slope’s steepness resulted in more fixations to the high side of the hole (van Lier et al., 2010). It indicated that the golfer fixates on a line on which the ball will roll. Usually, the purpose of golf putting is to put the ball into the hole with as few strokes as possible. However, the presence of a hole creates significant redundancy in the combination of angle and speed a golfer can choose. Thus, we set up a task wherein the ball would stop at the target and not in a hole, significantly reducing the angle and velocity tolerances. In this study, we asked the participants to show their aim point (aim direction) before hitting the ball to distinguish the strategy and the ensuing errors.

Once the aim direction was determined, the goal for the next phase was to align the clubface in a perpendicular direction to the target. This is the pre-motion phase and errors that occur in this phase are called pre-motion errors. The most critical issue in the pre-motion phase is that no errors occur between the aim direction and the clubface direction. For expert participants, we would presume that they would commit zero errors, but some studies have reported that even experts could not accurately align their clubface with the target direction (Johnston et al., 2003; van Lier et al., 2011). They also obtained inconsistent results regarding alignment error and proficiency. In addition, although studies were done on flat ground, alignment errors in slopes as a more practical situation have yet to be investigated. Therefore, we also examined alignment error as a pre-motion error that occurs in the pre-motion phase.

Many scholars have investigated errors during motion and results after golf putting. We will call these the motion and result errors, respectively. Backswing amplitude and impact velocity will influence ball roll distance (Mathers and Grealy, 2014), and clubface angle significantly affects directionality (Karlsen et al., 2008). Unskilled people showed higher movement variation than experts and had limited ability to control velocity and angle (e.g., Dias et al., 2014; Hasegawa et al., 2017). However, one problem in previous studies is that they have not fully explained whether this issue is a perceptual-level matter, a strategic-level matter, or a motor control matter. Therefore, this concern must be carefully considered, including kinematics. Despite the high correlation between backswing amplitude and clubhead impact velocity, the latter is more likely to account for ball roll distance (Hasegawa et al., 2019). Therefore, this research focuses on a golfer’s impact velocity and clubface angle. Studies show that the constant error (CE), variable error (VE), and absolute error (AE) in the final ball position (FBP) of unskilled people in the post-performance phase are worse than in the experts (e.g., Suzuki et al., 2019; Hasegawa et al., 2020). These results indicate the poor accuracy and precision of their movements. This study also intends to confirm the indicators following previous studies as result errors.

Considering the above, we recruited professional and amateur golfers to investigate the effects of proficiency and slope conditions. We divided the stages into the environmental perception phase, the decision-making phase, the pre-motion phase, the motion phase, and the post-performance phase and thoroughly investigated the errors that occur in each. We sought to determine the phase when errors occurred in a golf putting task, which requires accurate environmental perception and fine movement control. We specifically focused on environmental perception and strategy error, which remain a challenge in previous studies.

## Materials and Methods

### Participants

This study included 12 tour professionals and 12 amateur golfers whose average ages were 35.8 ± 6.1 and 47.5 ± 7.4 years, respectively, and whose average golfing experience was 24.2 ± 6.9 and 19.3 ± 9.1 years, respectively. There was a significant difference between the ages of professionals and amateurs (*F*_1,22_ = 17.71, *p* = 3.62 × 10^–4^, *f* = 0.90). The amateurs were intermediate players with an average score of 88.3 ± 2.5. The annual number of times played on the golf course was 132.5 ± 39.6 for professionals and 56.0 ± 29.2 for amateurs. The dominant hand in golf play of all participants was right hand, but in the dominant hand test, one amateur participant’s dominant hand was left and the other participants’ dominant hand was right. Moreover, all participants had normal or corrected-to-normal vision. Before participating, all participants provided written informed consent after a thorough explanation of the study. All experimental procedures were approved by the Ethics Committee of Iwate University and conformed to the principles of the Declaration of Helsinki.

### Task and Apparatus

**FIGURE 1:**
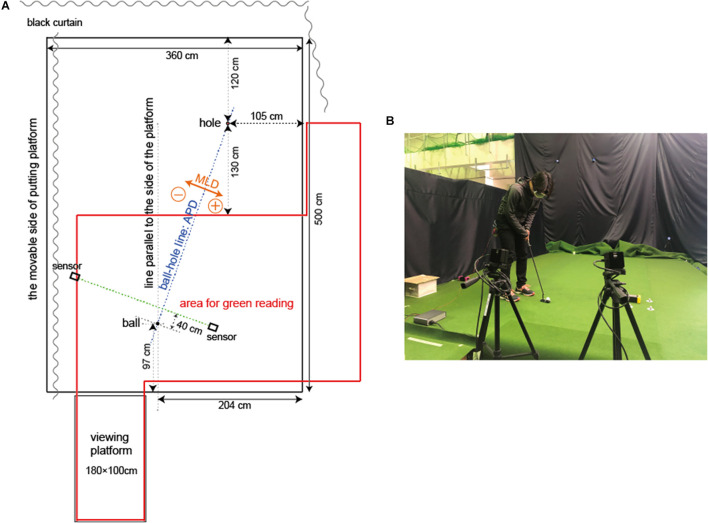
Experiment setting: **(A)** represents the pattern diagram of the experiment setting, while **(B)** shows a participant wearing shielded goggles for measurement. The experiment area where the putting platform was set was surrounded by curtains, and care was taken not to give the participants reference frames. The area surrounded by the red line indicates where participants could move freely to read the green. Two sensors were placed so that the shutter goggles would be activated when the ball passed the line 40 cm in front of where it was set. APD, anteroposterior direction; MLD, mediolateral direction.

The task employed a 3.0 m putting distance under each of the flat and 0.4-degree conditions. For the 0.4-degree condition, the left-to-right line (tilted to the right relative to the hitting direction) was set following the empirical finding that most golfers are not good at it compared to the right-to-left line (tilted to the left relative to the hitting direction). Participants played ten trials in each condition. Their goal was to stop a ball at the center of a 10.8 cm diameter hole (the same size as that in an actual golf green) drawn by a white magic marker on an artificial turf designed for putting practice (5.0 × 3.6 m) (Superbent, Newtons Inc., Kochi, Japan). The participants did not receive any explicit slope and distance information. Therefore, they were required to judge the presence or absence of a slope, set the clubhead to the appropriate direction, and exert the appropriate force. They verbally described the slope condition before starting the putting trials for each condition. In addition, before putting, the participants indicated their aim points from which the ball would be launched (see Procedure for more detail). All participants wore instant shielding goggles (AO-FOS, Applied Office Co., Ltd., Tokyo, Japan), limiting their field of view to 40 cm in front of the ball. The system became shielded whenever a ball crossed the light between the photoelectric sensors (see Figure 1). It was necessary to prevent learning during trials to confirm performance reproducibility. The goggle lens was transparent, and the field of view 6 m away was clear. Participants’ hearing was unobstructed and they could hear the impact of the ball. However, because the participants used an artificial turf designed for putting, they could not hear the ball rolling. The putting platform was metal, framed on a grid with robust wooden boards with attached artificial turf. Also, this platform was made to operate on one side (5.0 m) by electric winches (see also Figure 1).

Aim direction, clubhead kinematics, and ball trajectories were recorded by nine optical motion-capture cameras (Prime13, OptiTrack Japan, Ltd., Tokyo, Japan) operating at 240 Hz. We attached a reflective sheet to the ball to capture ball trajectory (see Supplementary Table 1 and Supplementary Figure 1 for more detail). The stimp rating, which indicates the speed of the putting green, is approximately 12 ft when the ball is used and is under the fast category. Also, 12 mm reflective markers were attached to the toe, heel, and neck of the putter head to digitize the club positions. The root mean square errors of the static and dynamic calibrations were <1.0 cm during all sessions. Calibration means the difference between the calculated and idealized coordinate values. All participants used the same putter (SB-01HB, PRGR Corp., Yokohama, Japan) and balls (Srixon Z-Star XV, Dunlop Sports Co., Ltd., Hyogo, Japan).

### Procedure

The flat and the 0.4-degree conditions were in random order and counterbalanced. In each one, the participants practiced with ten balls in a waiting area (the same product as the artificial turf installed for measurement) to familiarize themselves with the turf. In this familiarization session, we also asked the participants to wear shielding goggles. However, during practice, the participant’s field of vision was unobstructed after hitting. After the familiarization session, the participants moved to the experiment area with the researcher and received the following instructions while watching the actual putting platform. “From now on, please try to putt ten times here. Your goal is to stop the ball at the center of the hole. Please aim to hit the ball as close to the center of the hole as possible. It is the same distance as the putting distance you practiced earlier. But I cannot tell you what this putting line is. Feel free to move around for the next 3 min and read the putting line.” Subsequently, the researcher explained the green reading area to the participants (see Figure 1). Three minutes later, the researcher asked the participants three questions: “Is this putting line flat? Is the left side high? Is the right side high?” After responding, they put on instant shielding goggles. Then they were instructed how to set the ball and placed it in position by themselves. Afterward, they crouched behind the ball (toward the target) as they would in actual play and indicated their intended ball launch direction. At this time, the researcher moved the aim point marker while following the participant’s instructions and adjusted it until they thought it was in the correct position. The researcher recorded this marker position and then attached a circular white sticker (0.8 cm diameter) on the artificial turf at the same position as the aim point marker. In an actual golf green, golfers find an aim point in the direction they want to launch the ball and set up toward this spot. That is to say, golfers usually crouch behind the ball (toward the target) and find different color turf or scratches to clarify their aim point before hitting. Since the artificial turf did not have any discolorations (uniformly green), it was possible to prevent the participants from losing sight of the direction they were aiming for during the setup when the white sticker was attached. After this, the participants practiced only once to get used to their blocked field of vision immediately after hitting the ball. They were also told there was no time limit for hitting. They could incorporate their own routine. After the participants hit the ball, the researcher removed the white sticker. The participants repeated this process ten times after the phase when they indicated their aim to hit the ball. When the condition trial was over, participants removed the goggles and moved to the waiting area. They took a 15-min break and then tried another condition.

### Dependent Variables

**FIGURE 2:**
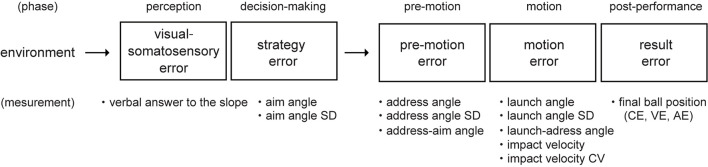
Schematic diagram showing the definition of errors in each phase and the study measurements. The golf putting activity was divided into five phases. The error definition and measurement variables in each phase are shown.

Figure 2 summarizes the errors in each golf putting phase and the dependent variables to measure the errors. The details present below.

#### Perception Phase

The participants responded (verbally) to three questions to measure their visual–somatosensory errors in the environmental perception phase: “Is this putting line flat? Is the left side high? Is the right side high?” Their responses were recorded by the researchers.

#### Decision-Making Phase

To identify strategy errors in the decision-making phase, the participants crouched behind the ball (toward the target) as they would in an actual play and indicated their intended ball launch direction. The aim direction (aim point) was determined as the angle with a line connecting the ball and the center of the hole as 0° (ball-hole line: see Figure 1).

#### Pre-motion Phase

To evaluate pre-motion errors in the pre-motion phase, the clubface orientation of the setup just before hitting (address angle) was calculated as 0° when the clubface was perpendicular to the ball-hole line. To determine the alignment error, the aim angle was subtracted from the address angle in each trial (address–aim angle), and the representative value for each individual was the average of ten trials. An address–aim angle of zero indicates that there is no alignment error. For all angles (including the ball launch angle described below), negative values indicate the left side of the ball-hole line, and positive values indicate the right side of the ball-hole line (see also Figure 1).

#### Motion Phase

To investigate motion errors in the motion phase, putting movement was divided into stages: backswing, downswing, ball impact, and follow-through (Couceiro et al., 2013; Dias and Couceiro, 2015). Because ball roll distance highly depends on impact velocity (Hume et al., 2005; Mathers and Grealy, 2014), the midpoint between the toe and heel of the clubhead was calculated to analyze the midpoint impact velocity. We defined impact velocity as the peak velocity of the clubhead (Hasegawa et al., 2017, 2019, 2020). We also assessed the ball launch angle, which we calculated using the direction of the launched ball, that is, the average angle for 1 s from 0.1 s after ball impact in the flat condition and the average angle for 0.2 s from 0.1 s after ball collision in the 0.4-degree condition (because the slope effect). To check the difference between the angle of the launched ball and the face angle of the clubhead just before the start of motion, the address angle was subtracted from the ball launch angle in each trial (launch–address angle), and the average of ten trials was used as the representative value for each participant.

All digitized data were smoothed with a fourth-order Butterworth filter (5 Hz cutoff) based on the root mean square of the residual error between the original and smoothed data (Jackson, 1979; Winter, 1990). Additionally, for aim angle (decision-making phase), address angle (pre-motion phase), impact velocity (motion phase), and ball launch angle (motion phase), the coefficient of intraindividual variation was calculated for impact velocity (impact velocity CV), and the standard deviation was calculated for each angle because intraindividual variation was an essential variable for motor learning (Dhawale et al., 2017). Since all angle values were close to zero, the variation coefficient was unsuitable to understand intraindividual variability. Therefore, we analyzed the standard deviation of the aim angle, address angle, and ball launch angle [aim angle standard deviation (SD), address angle SD, and launch angle SD, respectively].

#### Post-performance Phase

To examine the result errors in the post-performance phase, we analyzed the FBPs in terms of the anteroposterior (APD) and the mediolateral directions (MLD), and determined the CE, VE, and AE values. When the ball stopped at the hole’s center, the APD and MLD error values were zero.

### Statistics

To assess visual–somatosensory errors, the total numbers per answer for each condition (left side high, flat, and right side high) were counted, and chi-squared tests were conducted to compare skill levels. To investigate strategy errors, the relation between the two groups (professional and amateur) and two conditions (flat and 0.4°) were assessed using a two-factor mixed-design analysis of variance (ANOVA) for aim angle and aim angle SD.

To examine pre-motion errors, we conducted a two-factor mixed-design ANOVA (group × condition) for address angle and address angle SD. To investigate alignment errors for the address–aim angle, a three-factor mixed-design ANOVA was performed to explain the relationship among the two groups, the two conditions, and the two gaps [actual and ideal (i.e., 0)]. Condition and gap were repeated measures.

To analyze motion errors, we conducted a two-factor mixed-design ANOVA for impact velocity, impact velocity CV, launch angle, and launch angle SD. For the launch–address angle, to compare with zero, we also carried out a three-factor mixed-design ANOVA (group × condition × gap). For result errors (CE, VE, and AE), we also conducted a two-factor mixed-design ANOVA.

We also calculated “*f*” values as effect-size indices for the ANOVAs (Faul et al., 2007) and “Cramer’s V” values as effect-size indices for the chi-squared tests. According to Cohen’s (1988) conventions, small (*f* = 0.10), medium (*f* = 0.25), and large (*f* = 0.40) effect sizes were reported. All data were analyzed using PASW Statistics (ver. 18.0, IBM Japan, Ltd., Tokyo, Japan). The alpha level of significance was set to *p* < 0.05, but effect sizes above it were mentioned as well (one variable in this analysis).

## Results

### Visual–Somatosensory Error

**FIGURE 3:**
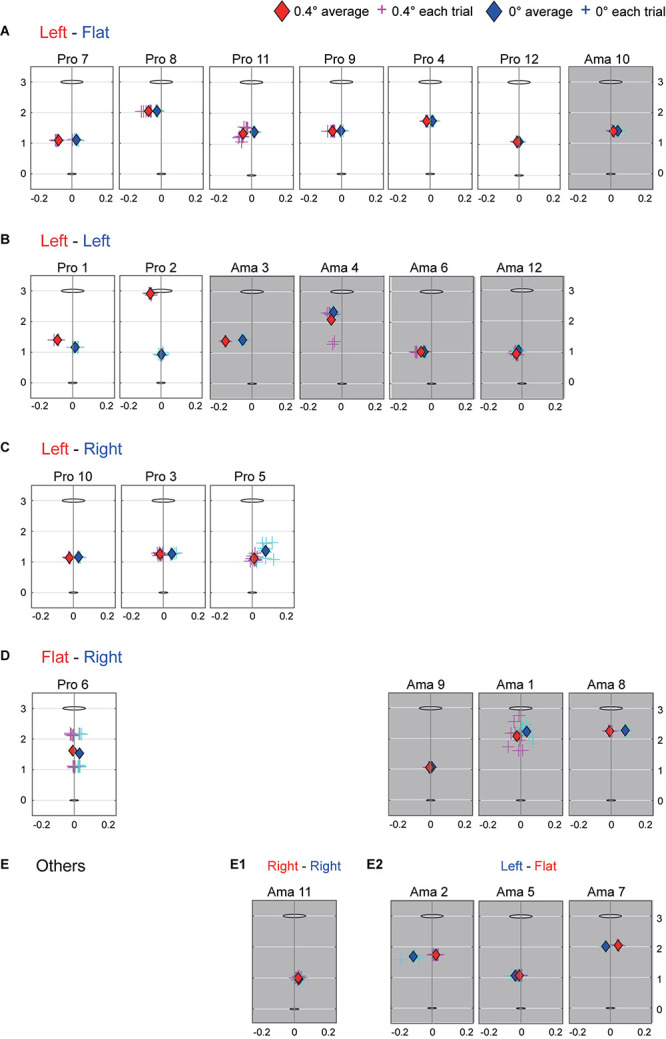
Participants’ slope perception and average aim point in the 0- and 0.4-degree conditions. The slope perception response was divided into five patterns from **(A–E)**. The red letters indicate answers in the 0.4-degree condition, while the blue letters indicate answers in the 0-degree condition. For example, in **(A)**, the correct answer pattern is shown in which the left is high in the 0.4-degree condition and flat in the 0-degree condition. In **(E)**, Ama 11 stated that the right side was high in both conditions, while Ama 2, 5, and 7 said that the left side was high in the 0-degree condition and flat in the 0.4-degree condition. Besides these slope perception responses, the participants’ aim points are shown. Both the vertical and horizontal axes for each participant show the distance (m). The coordinate (0, 0) ellipse is the ball, and the coordinate (0, 3) ellipse is the hole.

Figure 3 shows each participant’s answer regarding their slope perception in both conditions. Tables 1, 2 indicate the total number for the flat and 0.4-degree conditions, respectively. Chi-squared tests revealed significant differences in the flat condition [χ _(2)_ = 6.35, *p* < 0.05, Cramer’s V = 0.51, Table 1] and the 0.4-degree condition [χ _(2)_ = 6.82, *p* < 0.05, Cramer’s V = 0.53, Table 2]. The residual error of the flat condition revealed that more amateurs answered, “The left side is high,” and more professionals answered, “flat.” Meanwhile, the residual error of the 0.4-degree condition showed that more professionals answered, “The left side is high,” and more amateurs answered “flat,” This means that more professionals could perceive the slopes more accurately than amateurs.

**TABLE 1:** Total number of answers on slope perception for the 0-degree condition.

	**Left**	**Flat**	**Right**
Pro	2 ▽	6 ▲	4
Ama	7 ▲	1 ▽	4

*△▼ mean the results of the residual analysis.*

**TABLE 2:** Total number of answers on slope perception for the 0.4-degree condition.

	**Left**	**Flat**	**Right**
Pro	11 ▲	1 ▽	0
Ama	5 ▽	6 ▲	1

*△▼ mean the results of the residual analysis.*

### Strategy Error

**FIGURE 4:**
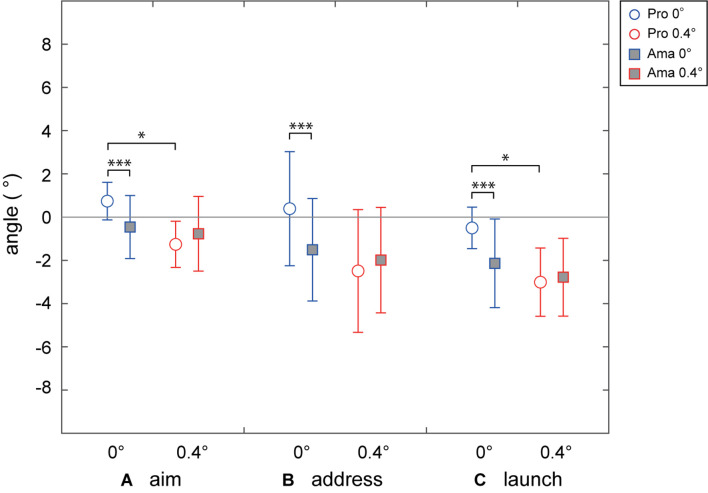
Average angles of each phase **(A–C)** in the 0- and 0.4-degree conditions. The angle 0 refers to the center of the target. Negative values indicate the position to the left of the target’s center, while positive values indicate the position to the right of the target’s center. *p < 0.05 and ***p < 0.001.

**FIGURE 5:**
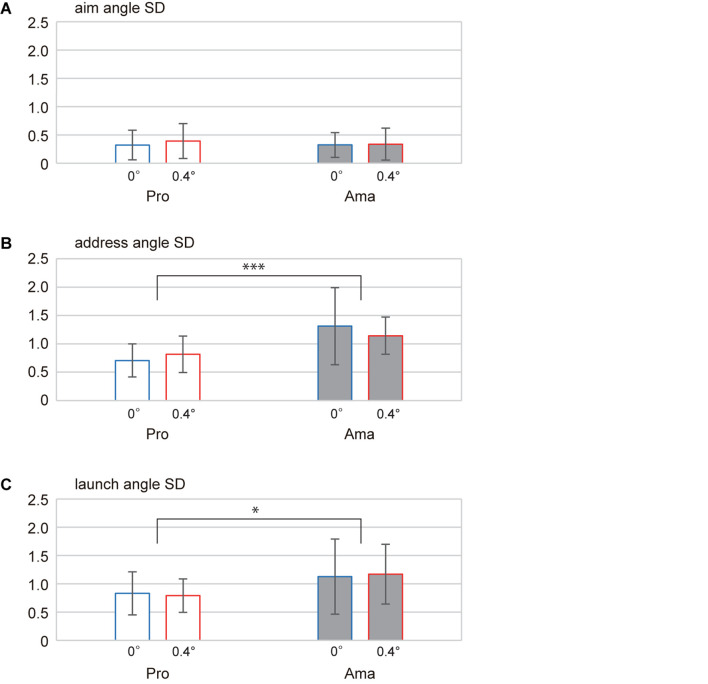
Average angle SDs for each phase in the 0- and 0.4-degree conditions. These values showed the intraindividual variability of face angles for each phase and were averaged for each group. *p < 0.05 and ***p < 0.001.

Figure 3 shows each participant’s average aim point in both conditions. Figures 4A, 5A indicate the average aim angles and aim angle SDs of both groups for each condition. The two-way ANOVA results for aim angle showed a significant interaction (*F*_1,22_ = 7.45, *p* = 0.012, *f* = 0.58). Simple-effects testing indicated that the aim angles of both groups were significantly different in the flat condition (*F*_1,22_ = 5.46, *p* = 0.029, *f* = 0.50) and that the aim angles of professionals were significantly different depending on the condition (*F*_1,22_ = 20.85, *p* = 1.51 × 10^–4^, *f* = 0.97). In the flat condition, the aims of both groups were different, with amateurs favoring the left side more than professionals. Also, the main effect of condition was significant (*F*_1,22_ = 13.90, *p* = 0.001, *f* = 0.79), but that of group was not. The results of the two-way ANOVA for aim angle SD revealed a non-significant interaction, and the main effects of group and condition were not significant as well. Table 3 shows the average aim angles of the hole-in trials as the correct aiming angle.

**TABLE 3:** Average ball launch angles and impact velocities in the hole-in trials.

	**0°**		**0.4°**	
	**Angle**	**Velocity**	**Angle**	**Velocity**
Average	0.00	1.24	−6.50	1.19
SD	0.75	0.05	1.07	0.04
*n*	37 (7)		27 (1)	

*The angle and velocity are the mean and standard deviation (SD) of the launch angle and impact velocity calculated from the number of trials indicated by n. For n, the figures in parentheses refer to the number of hole-in trials measured during the experiment. The other trials used hole-in trials by professionals with visual feedback. The unit for angle is °, while the unit for velocity is m/s.*

### Pre-motion Error

Figures 4B, 5B indicate the average address angles and address angle SDs of both groups for each condition. The results of the two-factor ANOVA for address angle showed a significant interaction (*F*_1,22_ = 10.71, *p* = 0.003, *f* = 0.70). According to simple-effects testing, the address angles of the professionals were significantly different depending on the condition (*F*_1,22_ = 30.87, *p* = 1.38 × 10^–5^, *f* = 1.18). Also, the main effect of condition was significant (*F*_1,22_ = 21.03, *p* = 1.43 × 10^–4^, *f* = 0.98), while that of group was not. Meanwhile, the two-way ANOVA results for address angle SD revealed that the interaction and main effects of the condition were not significant. However, the main effect of the group was significant (*F*_1,22_ = 10.40, *p* = 2.86 × 10^–12^, *f* = 0.69); the address angle SD of amateurs was larger than that of professionals.

**FIGURE 6:**
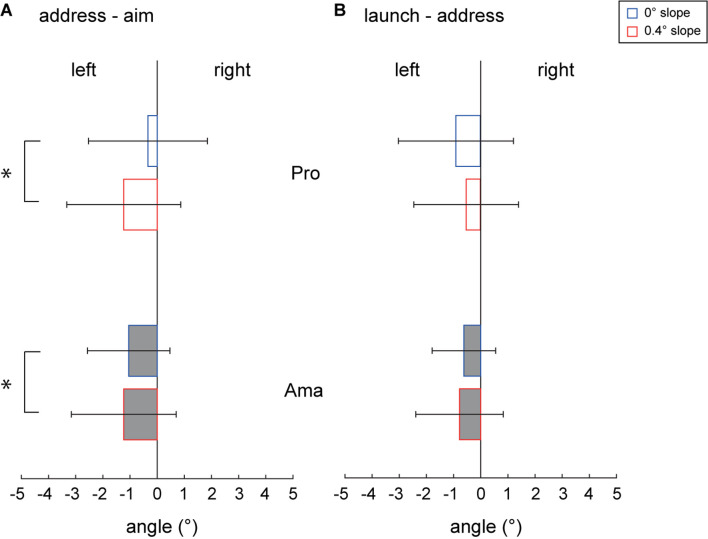
Average alignment error in the pre-motion phase and average motion error in the motion phase. The address-aim angle **(A)** indicates the average of each group of values obtained by subtracting the aim angle from the address angle. The launch-address angle **(B)** indicates the average of each group of values obtained by subtracting the ball launch angle from the address angle. All error bars indicate ±1SD. *p < 0.05.

Figure 6A shows the differences between the aim and address angles of both groups for each condition. The three-factor ANOVA results for address–aim angle revealed that the second-order interaction (group × condition × gap) was not significant. However, a significant first-order interaction was observed (condition × gap, *F*_1,22_ = 5.03, *p* = 0.035, *f* = 0.48). Additionally, simple-effects testing indicated that address–aim angle in the 0.4-degree condition was larger than that in the flat condition (*F*_1,22_ = 5.03, *p* = 0.035, *f* = 0.48), and the address–aim angle in the 0.4-degree condition was significantly different from zero (*F*_1,22_ = 8.16, *p* = 0.009, *f* = 0.61). Also, the main effects of condition (*F*_1,22_ = 5.03, *p* = 0.035, *f* = 0.48) and gap (*F*_1,22_ = 5.83, *p* = 0.025, *f* = 0.51) were significant. However, the main effect of the group was not significant. Therefore, no skill level differences were observed in address–aim angle, and the address–aim angle of the 0.4-degree condition was larger than that of the flat condition. In addition, the clubface was oriented toward the left side of the aim, which was significantly different from 0 and particularly remarkable in the 0.4-degree condition.

### Motion Error

Figures 4C, 5C show the average launch angles and launch angle SDs of both groups for each condition. The two-way ANOVA results for launch angle revealed a significant interaction (*F*_1,22_ = 6.28, *p* = 0.002, *f* = 0.53). Simple-effects testing results showed significant differences in the launch angle of both groups in the flat condition (*F*_1,22_ = 5.67 *p* = 0.026, *f* = 0.51) and in the launch angle of professionals depending on the conditions (*F*_1,22_ = 22.64, *p* = 9.48 × 10^–5^, *f* = 1.01); in the flat condition, the launch angle of both groups was different, with amateurs inclined toward the left side more than professionals. In addition, the main effect of condition was significant (*F*_1,22_ = 17.83, *p* = 3.50 × 10^–4^, *f* = 0.90), but that of group was not. The two-way ANOVA results for launch angle SD revealed non-significant interaction and main effects of the condition. However, the main effect of group was significant (*F*_1,22_ = 4.44, *p* = 0.047, *f* = 0.45); the launch angle SD of amateurs was larger than that of professionals.

Figure 6B presents the differences between the address and launch angles of both groups for each condition. The three-factor ANOVA results for launch–address angle revealed that the second-order and first-order interactions and all main effects were not significant. Therefore, launch–address angle had no differences in skill level, no differences due to condition, and no apparent differences from 0.

**FIGURE 7:**
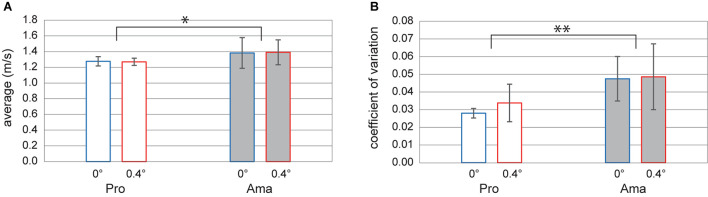
Averages of impact velocity and impact velocity CV. **(A)** Consists of the average values of the impact velocity for each group. **(B)** Composed of the average values of the variation coefficient in each group. The error bars indicate ±1SD. *p < 0.05 and **p < 0.01.

Figure 7 shows the impact velocity and impact velocity CV averages. The two-way ANOVA results for impact velocity revealed non-significant interaction and main effects of the condition. However, the main effect of group was significant (*F*_1,22_ = 4.40, *p* = 0.048, *f* = 0.45), and the impact velocity of amateurs was greater than that of professionals. Meanwhile, the results of the two-way ANOVA for impact velocity CV revealed that the interaction and main effects of the condition were not significant. However, the main effect of group was significant (*F*_1,22_ = 13.22, *p* = 0.001, *f* = 0.78), and the impact velocity SD of amateurs was greater than that of professionals.

**FIGURE 8:**
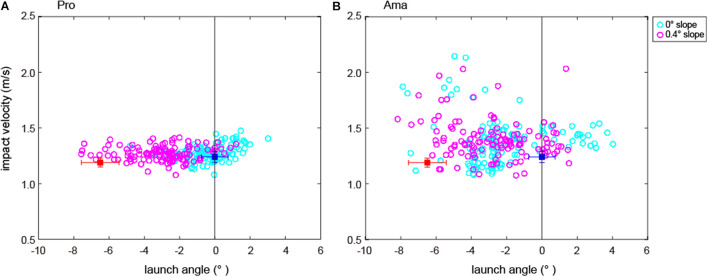
Relations between the impact velocity and launch angle of all participants’ trials and hole-in trials. **(A,B)** The plots of all trials (240 trials) involving ball launch angle and impact velocity for the 12 people in each group. The red and blue squares show the average and SD (±1SD) of the hole-in trials in the 0.4- and 0-degree conditions, respectively. The red and blue squares in **(A,B)** have the same values (see also Table 3 for details regarding the hole-in trials).

Table 3 shows the average impact velocities of the hole-in trials as the correct impact velocity, and Figure 8 shows the relations between launch angles and impact velocities in all trials.

### Result Error

#### Constant Error Assessment

**FIGURE 9:**
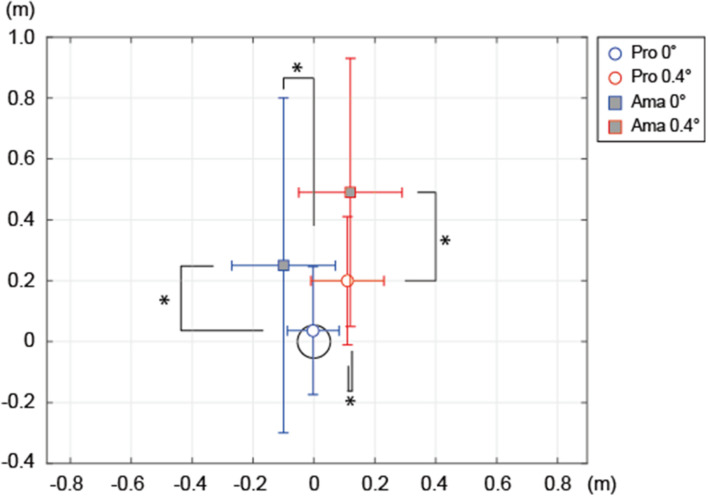
Average constant errors in final ball position for each group. The circle in coordinate (0, 0) represents the target. Both the vertical and horizontal axes indicate the distance (m). The positive values on the horizontal axis mean that the FBP was to the right of the target’s center, while the negative values indicate that the FBP was to the left of the target’s center. The positive values on the vertical axis signify that the FBP was overshot from the target’s center, while the negative values on the vertical axis indicate that it was undershot. *p < 0.05.

Figure 9 shows the average constant error (CE) of each group in both conditions. The two-way ANOVA results for CE in terms of anteroposterior direction (APD) revealed that the interaction and main effects of group were not significant. However, the main effect of condition was significant (*F*_1,22_ = 16.93, *p* = 4.57 × 10^–4^, *f* = 0.88), and CE APD was larger in the 0.4-degree condition than in the flat condition. Meanwhile, the two-way ANOVA results for CE in terms of mediolateral direction (MLD) revealed a significant interaction (*F*_1,22_ = 4.43, *p* = 0.047, *f* = 0.45). Simple-effects testing indicated that both groups (professionals: *F*_1,22_ = 9.23, *p* = 0.006, *f* = 0.65; amateurs: *F*_1,22_ = 36.16, *p* = 4.72 × 10^–6^, *f* = 1.28) were significantly different between conditions; that is, regardless of skill level, CE MLDs in both groups were larger at the 0.4-degree condition. Also, the main effect of condition was significant (*F*_1,22_ = 40.96, *p* = 1.94 × 10^–6^, *f* = 1.36).

#### Variable Error Assessment

Table 4 shows the variable error (VE) results. The two-way ANOVA results for VE in terms of APD revealed that the interaction and group (*F*_1,22_ = 3.58, *p* = 0.072, *f* = 0.40) were not significant. However, the main effect of the condition was significant (*F*_1,22_ = 4.36, *p* = 0.048, *f* = 0.44); VE MLDs in both groups were higher in the 0.4-degree condition regardless of skill level. The results of the two-way ANOVA for VE via MLD revealed that the interaction and the main effects of group and condition were not significant.

**TABLE 4:** Average variable errors in final ball position.

		**0°**		**0.4°**	
		**Average**	**SD**	**Average**	**SD**
APD	Pro	0.17	0.05	0.21	0.07
	Ama	0.22	0.07	0.25	0.08
MLD	Pro	0.09	0.04	0.10	0.04
	Ama	0.09	0.06	0.11	0.04

*SD, standard deviation; APD, anteroposterior direction; MLD, mediolateral direction. The unit is m.*

#### Absolute Error Assessment

Table 5 shows the results for absolute error (AE). The two-way ANOVA for AE via APD revealed a non-significant interaction and condition. However, the main effect of group was significant (*F*_1,22_ = 12.95, *p* = 0.002, *f* = 0.77); the AE APD of amateurs was greater than that of professionals. Meanwhile, the two-way ANOVA for AE in terms of MLD revealed that the interaction and main effects of the condition were not significant (*F*_1,22_ = 3.85, *p* = 0.062, *f* = 0.42). However, the main effect of group was significant (*F*_1,22_ = 11.62, *p* = 0.003, *f* = 0.73). The AE APD of amateurs was greater than that of professionals.

**TABLE 5:** Average absolute errors in final ball position.

		**0°**		**0.4°**	
		**Average**	**SD**	**Average**	**SD**
APD	Pro	0.22	0.12	0.28	0.17
	Ama	0.53	0.29	0.60	0.31
MLD	Pro	0.10	0.04	0.17	0.05
	Ama	0.19	0.08	0.21	0.10

*SD, standard deviation; APD, anteroposterior direction; MLD, mediolateral direction. The unit is m.*

Stepwise multiple regression analysis was performed as a complementary assessment to sequentially identify slope perception answers, aim angle, address angle, launch angle, and impact velocity (independent variables) to explain the APD of CE and the MLD of CE (dependent variables). Results showed that only the professionals’ MLDs in the 0.4-degree condition were less fit for modeling, but the goodness of fit of the other models was high. Therefore, we confirmed that impact velocity is the strongest predictor of the APD of FBP, and launch angle is the strongest predictor of the MLD of FBP. We also found that all models, except for the 0.4-degree condition of the professionals, significantly explained slope perception (more details in Supplementary Text Section “Stepwise Multiple Regression Analysis for the APD and MLD of CE”). Further, Table 6 presents the main results of this study.

**TABLE 6:** Results list of this study.

Errors in each phase	Results		
**Visual-somatosensory error**			
The number of collect answer to the slope	flat: ama < pro		
	0.4: ama < pro		
	**Interaction**	**Main effect of group**	**Main effect of condition**
**Strategy error**			
Aim angle	pro: flat > 0.4	n.s.	n.s
	flat: ama < pro		
Aim angle SD	n.s.	n.s.	n.s
**Premotion error**			
Address angle	pro: flat > 0.4	n.s.	flat > 0.4
Address angle SD	n.s.	ama > pro	n.s.
**Motion error**			
Launch angle	pro: flat > 0.4	n.s.	flat > 0.4
	flat: ama < pro		
Launch angle SD	n.s.	ama > pro	n.s.
Impact velocity	n.s.	ama > pro	n.s.
Impact velocity CV	n.s.	ama > pro	n.s.
**Result error**			
CE APD	n.s.	n.s.	flat > 0.4
CE MLD	pro: flat > 0.4	n.s.	flat > 0.4
	ama: flat > 0.4		
VE APD	n.s.	ama > pro	n.s.
VE MLD	n.s.	ama > pro	n.s.
AE APD	n.s.	ama > pro	n.s.
AE MLD	n.s.	ama > pro	n.s.

## Discussion

This study sought to identify emerging skill differences in the performance of a motor task requiring accurate environmental perception and fine movement control and pinpoint the phase where errors occur. We assigned golf putting as the task and divided it into several phases: the environmental perception phase, the decision-making phase, the pre-motion phase, the motion phase, and the post-performance phase. Next, we comprehensively examined the errors that occur in each one (Figure 2). We recruited 12 tour professional golfers and 12 intermediate golfers, who participated by putting on two surface conditions: flat and 0.4-degrees. The results showed differences between the professional and amateur groups in the environmental perception phase.

Regarding visual–somatosensory errors in the environmental perception phase, the number of correct answers for amateurs was significantly fewer in both conditions than for professionals (Tables 1, 2). In the flat condition, the correct answer percentage was not so high for professionals. However, in the 0.4-degree condition, 11 out of 12 professionals perceived that the putting line was high on the left side (i.e., break to the right, Figures 3A–C). As shown in Figures 3D,E, for seven amateurs, the situation in which their toes were down was either “flat” or “high on the right side.” Two perspectives explain these results. First, the amateur players’ perceptual systems may have poor tuning ability compared to the trained professionals. Motor learning is associated with systematic changes in proprioception (e.g., Haith et al., 2008; Ostry et al., 2010; Wong et al., 2012), and improves sensory acuity to generate accurate movements (Wong et al., 2011). Second, the amateurs could not find the information they needed from the environment. A previous study using a virtual golf putting task to examine participants’ green reading and slope perception found accuracy rates of 57% for amateurs and 76% for professionals (Campbell and Moran, 2014). Golfers’ eye gaze patterns and verbal reports were recorded as they read the slope of a virtual golf green from six different positions. Campbell and Moran (2014) suggested that distinctive periods of visual perceptual-cognitive attention may underpin higher levels of putting skill. Specifically, even when individuals look at the same screen, there will be differences in the method of extracting information, and the extracted data will vary. Another study reported that amateurs have lower motor control resolution than professionals (Hasegawa et al., 2017). Therefore, our results suggest that amateurs show lower perceptual resolution than professionals. In this study, we focused primarily on skill differences among participants. However, there was a significant age difference between the groups. Previous studies highlighted that proprioceptor differs depending on individual factors other than skill differences, such as age (Liutsko et al., 2014), gender (Liutsko et al., 2020), and cultural differences (Liutsko, 2019). Therefore, these problems are issues for future study.

To examine the participants’ strategy errors, we assigned them the challenge of stopping the ball in a circle without making a hole. This setting significantly reduces speed control tolerance and, inevitably, aiming angle tolerance. First, in the decision-making phase, we confirmed that all golfers set their aim point to the side in varying degrees as they perceived in the perception phase (Figure 3). Measuring this aim angle allows us to determine how well they perceived slope strength. The results showed that the aim of both groups was significantly different in the flat condition (Figure 4). The amateurs aimed to the left relative to the aims of the professionals. The professionals’ aim varied, depending on the slope conditions. However, depending on the condition, the aim of the amateurs was not significantly different. The primary cause of the differences in aiming direction between the professionals and amateurs was their visual–somatosensory error in the previous phase. That is, 7 of 12 amateurs misperceived the 0- and 0.4-degree conditions (Figures 3D,E), therefore, their aim was not significantly different depending on the condition. The result was no surprise, as experts are generally and consistently faster and more accurate than novices in various perceptual and cognitive paradigms (Starkes and Ericsson, 2003; Hodges et al., 2006; Mann et al., 2007), but our study clearly showed the link between perception and strategy error. We also confirmed that the amateurs’ aiming angle was insignificant between the two conditions because they dragged the visual–somatosensory error in the previous phase. However, these results do not indicate whether the professionals had an accurate aim direction for this experimental task. We will discuss later whether these experts’ decision-making was correct.

As for pre-motion errors, clubface address angle analysis revealed that the face angle at the address of the professionals depends on the slope conditions. It was unclear for amateurs (Figure 4). Thus, these results were also a continuation of the errors in previous phases. In addition, the address angle SD analysis showed that amateurs could not precisely turn to face the direction they wanted to aim (Figure 5B). Furthermore, when discussing pre-motion errors in golf, we must not dismiss alignment errors (Johnston et al., 2003; van Lier et al., 2011; Shim et al., 2019). Ideally, the address–aim angle should be zero, but this was distinctly different from 0 in both professionals and amateurs, especially in the 0.4-degree condition (Figure 6A), and their clubface oriented toward the left. In addition, we found a further increase in alignment errors in the slope condition, a new finding complementary to previous research.

Studies suggest that alignment errors, called systematic alignment errors or perceptual alignment bias, are associated with visual illusions. Johnston et al. (2003) and Shim et al. (2019) observed that golfers, regardless of skill, made alignment errors when perceiving the direction of the aim line between the ball and the hole (rightward bias). The results from these studies oppose the leftward bias in our study. Meanwhile, consistent with the present results, van Lier et al. (2011) experiment 3 and Karlsen et al. (2008) reported that the experts’ clubface was oriented about 1° to the left of the target. Although novices have a rightward bias (Johnston et al., 2003; van Lier et al., 2011; Shim et al., 2019), the intermediate players in our study did not have this tendency and were not significantly different from the professionals.

It was unclear why the alignment errors occurred. One study found that head position was associated with alignment error but did not specify whether it was the only cause (van Lier et al., 2011). Furthermore, a novelty in this study was its finding that alignment error increased when the putting surface inclined slightly. It may be because the participants’ toes-down posture affected their attitude control (Sasagawa et al., 2009) and therefore changed the position pattern of their heads and bodies, a theory emphasized by van Lier et al. (2011). Further research is necessary on this point. Considering the combined results from previous research and this study, it is almost certain that not only novices and amateur golfers but also professional golfers commit alignment errors. However, as mentioned in van Lier et al. (2011), the direction and magnitude of the error are considered skill- and task-dependent. Since the break-to-left slope condition (i.e., toes-up) was not set in this study, future studies would benefit from examining the relationship between the slope and alignment error after adding new conditions.

As for motion errors, we narrowed down the measurement items to impact velocity and ball launch angle. We confirmed that CE APD and CE MLD can be thoroughly explained by impact velocity and launch angle via stepwise multiple regression analysis, respectively (Supplementary Text Section “Stepwise Multiple Regression Analysis for the APD and MLD of CE”). Amateurs’ impact velocity was faster than that of professionals, and their impact velocity CV was greater than that of professionals (Figure 7). These results support previous studies (e.g., Hume et al., 2005; Hasegawa et al., 2020). However, our results did not clarify whether the amateurs felt the target was far away or solely could not appropriately control their force. We also found variations in the professionals’ ball launch angle depending on slope conditions. In the flat condition, the ball launch angles of both groups differed, with those of the amateurs favoring the left compared with the professionals (Figure 4). These results also follow the errors of previous phases. Moreover, the fact that the amateurs’ ball launch angle SD was greater than that of the professionals indicates that the amateurs’ face control precision was also poor. To clarify whether unequivocal differences exist between the direction of the ball held at the address and the one at actual launch, we further examined the address–launch angle, observing no significant differences from 0 and no significant differences between groups and conditions. It suggests that the golfers compensated for their alignment and pre-motion errors during the motion phase.

Furthermore, to understand whether the professionals were making the correct decisions, we calculated the average value of the ball launch angle and the impact velocity within the target stop (Table 3). Figure 8 clearly shows that the amateurs’ launch angles and velocities were almost far from the correct answer values compared with those of the professionals and that only a few professional trials were within the vicinity of the correct answer values at the 0.4-degree condition. Simply put, even professionals were committing strategy errors. This result is also consistent with Pelz (1994), which found that all participants systematically underestimate the break of putts, reporting an average of just 25% of the true break regardless of skill level. We believe that these results can be explained from three points of view. First, the familiarization session in our study was conducted in a different place (waiting area) that had the same type of artificial turf (see also Figure 1); however, there was a downhill slope in 0.4-degree condition because of the angled positional relation between the ball and the hole. Thus, the ball rolled faster as opposed to the familiarization session. Second, the area where the participants could read the green was limited. For example, they could not read the line from the other side of the hole, restricting the required information to understand the environment. Third, since golf play includes a hole component, the redundancy in velocity control must be recognized. We recognize the extreme difficulty, even for professionals, in planning the angle and speed to stop a ball at a 10.8 cm hole from a 3 m distance without visual feedback. However, we are convinced that the experiment had the appropriate setting to understand which phases were hindering the amateurs’ progress.

The resulting error, constant error (CE), variable error (VE), and absolute error (AE) of the final ball position (FBP) were analyzed separately for anteroposterior (APD) and mediolateral directions (MLD). The CE assessment for APD and MLD confirmed no significant differences between the groups and that the CE of both groups is larger in the 0.4-degree condition than in the flat condition (Figure 9). Also, in terms of AE (Table 5), both the CE APDs and MLDs of the amateurs were larger than those of the professionals. For VE assessment (Table 4), the amateurs’ APDs tended to be larger than those of the professionals, which could be explained by the large variability in the amateurs’ impact velocities because of the high correlation between impact velocity and the APD of FBP (e.g., Hasegawa et al., 2019). As in previous studies (Johnston et al., 2003; van Lier et al., 2011; Shim et al., 2019), there were no significant indications that the FBPs were biased depending on alignment error, so we did not confirm whether perceptual alignment error affected result error especially in the flat condition.

To understand the results of the seven amateurs who misperceived the slopes, we checked the individual results focusing on CE (Supplementary Table 2). The sorting data showed that most of these participants ranked worse (far from the center of the hole), especially in the 0.4-degree condition. In particular, Ama 1, Ama 2, Ama 7, Ama 8, and Ama 11 provided the wrong answers for slope perception and then aimed for the straight or right side of the hole in the 0.4-degree condition even though it was a break to the right. Therefore, one would reasonably presume that the extreme right-side mistake in the 0.4-degree condition was attributable to the perception phase. Moreover, Ama 5 answered “flat” in the 0.4-degree condition and showed a straight aim, but their averaged FBP was to the left side of the hole, which may be due to problems with his/her movement. In addition, stepwise multiple regression analyses showed that slope perception significantly influenced the result errors in almost all models. From the above, our results showed that intermediate-level amateurs were unable to adjust the velocity control of the clubhead to the appropriate level and that their clubhead velocity and clubface angle control were less reproducible than those of professionals in the motion phase. Furthermore, we believe that the amateurs’ pre-motion and strategy errors depended on their visual–somatosensory errors. In Schmidt’s information-processing model, each phase is serial—that is, if the first phase is incorrect, the following ones would be wrong. Therefore, following this model, we suggest that the visual–somatosensory errors of intermediate amateurs serve as obstacles to their progress.

There are several models of human error (Tous-Ral and Liutsko, 2014). We analyzed the errors in each phase based on Schmidt’s models which are used commonly in the field of motor learning research. As limits of our study, there was a significant difference between the ages of professionals and amateurs. Previous studies showed that proprioceptor varies depending on age (Liutsko et al., 2014), gender (Liutsko et al., 2020), and cultural differences (Liutsko, 2019). This study did not rule out these factors. Furthermore, we examined only the break-to-right slope (i.e., toes-down), but the break-to-left slope (i.e., toes-up) was not set, so future research must also conduct experiments that can clarify the relation between systematic perceptual errors and green slopes, which was a persistent issue in this research. We suggest that perceptual errors lead to errors in the next phase in our results. However, motion error is also affected by neuromotor noise (Dhawale et al., 2017).

## Conclusion

We divided the golf putting task into five phases from environmental perception to post-performance, thoroughly examined the errors that occurred in each, and comprehensively explained their associations. Our results revealed that while many professionals perceived subtle differences in the environment, more than half of the amateurs committed visual–somatosensory errors in the perception phase. Examining the errors in each phase revealed that the errors of the post-performance phase were linked retroactively to the errors in the motion phase, pre-motion phase, decision-making phase, and perception phase. Based on Schmidt’s information-processing model, we suggest that the visual–somatosensory errors of intermediate amateurs serve as obstacles to their progress. Therefore, it became clear that differences in discrete motor skill levels emerge from the environmental perception stage. To the best of our knowledge, studies that investigate perceptual and strategic errors while carefully examining kinematics are rare. We believe that the knowledge gained from our research can be applied to future motor learning studies and the sports universe.

## Data Availability Statement

The datasets presented in this study can be found in online repositories. The names of the repository/repositories and accession number(s) can be found in the article/Supplementary Material.

## Ethics Statement

The studies involving human participants were reviewed and approved by the Iwate University. The patients/participants provided their written informed consent to participate in this study.

## Author Contributions

YH, AO, and KF conceived and designed the experiments. YH and AO conducted the experiments. YH and KF analyzed the data. All authors contributed to the article and approved the final manuscript.

## Conflict of Interest

The authors declare that the research was conducted in the absence of any commercial or financial relationships that could be construed as a potential conflict of interest.

## Publisher’s Note

All claims expressed in this article are solely those of the authors and do not necessarily represent those of their affiliated organizations, or those of the publisher, the editors and the reviewers. Any product that may be evaluated in this article, or claim that may be made by its manufacturer, is not guaranteed or endorsed by the publisher.
