# Supplementary material for: Skill Differences in a Discrete Motor Task Emerging From the Environmental Perception Phase
Source: Front Psychol. 2021 Oct 1;12:697914. doi: 10.3389/fpsyg.2021.697914 (PMC8517186; doi:10.3389/fpsyg.2021.697914)
Supplement: Supplementary file 1 [file Data_Sheet_1.DOCX]

Supplementary Material

# Supplementary Text

## Stepwise Multiple Regression Analysis for the APD and MLD of CE

As additional analyses, stepwise multiple regression analysis was performed to sequentially identify slope perception answers, aim angle, address angle, launch angle, and impact velocity (independent variables) to explain the APD and MLD of CE (dependent variables). This was carried out for each group and condition, for a total of eight times. The slope perception answers, which were a nominal-scale variable, were converted into dummy variables (˗1: left side high, 0: flat, 1: right side high). Standardized partial regression coefficients (β) and coefficients of determination were obtained. F-value was also used to check whether the model was statistically significant. In addition, we checked the correlation matrix table, but since no variables would result in |r| > 0.8, we targeted all variables. The variance inflation factor of all statistics was less than 10.0, and no multicollinearity was observed. We performed these stepwise multiple regression analyses using PASW Statistics (ver. 18.0, IBM Japan, Ltd., Tokyo, Japan).Supplementary material is not typeset so please ensure that all information is clearly presented, the appropriate caption is included in the file and not in the manuscript, and that the style conforms to the rest of the article. To avoid discrepancies between the published article and the supplementary material, please do not add the title, author list, affiliations or correspondence in the supplementary files.

**1.1.1. Results for Professionals**

Approximately 70 % of the variability in the flat-condition CE APD of the professionals was significantly explained by four variables (F4, 119 = 66.65, p = 4.60 × 10˗29): impact velocity ((β) =.903, p = 1.60 × 10˗25), aim angle ((β) = ˗.308, p = 3.27 × 10˗5), launch angle ((β) = ˗.196, p =.002), and perception ((β) =.219, p = 002). We confirmed that the strongest predictor of CE APD of the professionals was impact velocity. Three variables (F3, 119 = 89.27, p = 5.26 × 10˗30), which were launch angle ((β) = 1.00, p = 1.76 × 10˗30), perception ((β) = ˗.306, p = 9.73 × 10˗7), and address angle ((β) = ˗.160, p =.009), combined to significantly explain approximately 70% of the variance in the flat-condition CE MLD of the professionals. We confirmed that the strongest predictor of CE MLD of the professionals was launch angle.

Approximately 63% of the variability in the 0.4-degree-slope CE APD of the professionals was significantly explained by three variables (F3, 119 = 66.08, p = 5.46 × 10˗25): impact velocity ((β) =.657, p = 3.20 × 10˗18), address angle ((β) = ˗.265, p = 9.32 × 10˗6), and perception ((β) = ˗.233, p = 3.79 × 10˗4). We confirmed that the strongest predictor of CE APD of the professionals was impact velocity. Two variables (F2, 119 = 36.41, p = 5.10 × 10˗13), which were launch angle ((β) =.598, p = 2.92 × 10˗13) and impact velocity ((β) = ˗.191, p =.010) combined to significantly explain approximately 38% of the variance in the 0.4-degree-slope CE MLD of the professionals. We confirmed that the strongest predictor of CE MLD of the professionals was launch angle.

**1.1.2 Results for Amateurs**

Approximately 75% of the variability in the 0-degree CE APD of the amateurs was significantly explained by two variables (F2, 119 = 173.10, p = 1.10 × 10˗35): impact velocity ((β) =.611, p = 6.81 × 10˗24) and perception ((β) =.482, p = 1.58 × 10˗17). We confirmed that the strongest predictor of CE APD of the amateurs was impact velocity. Three variables (F3, 119 = 230.14, p = 1.15 × 10˗48), which were launch angle ((β) =.814, p = 1.34 × 10˗31), perception ((β) =.149, p =.005), and impact velocity ((β) = ˗.079, p =.040), combined to significantly explain approximately 86% of the variance in the flat-condition CE MLD of the amateurs. We confirmed that the strongest predictor of CE MLD of the amateurs was launch angle.

Approximately 65% of the variability in the 0.4-degree-slope CE APD of the amateurs was significantly explained by two variables (F2, 119 = 110.38, p = 1.16 × 10˗27): impact velocity ((β) =.794, p = 1.26 × 10˗27) and perception ((β) =.340, p = 1.09 × 10˗8). We confirmed that the strongest predictor of CE APD of the amateurs was impact velocity. Two variables (F2, 119 = 69.37, p = 1.36 × 10˗20), which were launch angle ((β) =.454, p = 2.25 × 10˗8] and perception ((β) =.378, p = 2.14 × 10˗6), combined to significantly explain approximately 54% of the variance in the 0.4-degree-slope CE MLD of the amateurs. We confirmed that the strongest predictor of CE MLD of the amateurs was launch angle.

Hence, only the professionals’ MLDs in the 0.4-degree slope showed less fit for modeling, but the goodness of fit of the other models was high. Therefore, we confirmed that the strongest predictor of the APD of FBP was impact velocity and that the strongest predictor of the MLD of FBP was launch angle. We also found that all models significantly explained slope perception, except for the 0.4-degree condition of the professionals.

# Supplementary Figure and Tables

## Supplementary Figure

**
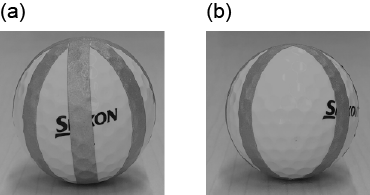
**

# Supplementary Figure 1. Ball with a Reflective Sheet. The ball was set so that the lines of the reflective sheet were vertical. (a) A sheet with a width of 5 mm was attached to the ball as shown in (b). Sheets that were hit by the club head were excluded.

## Supplementary Tables

**Supplementary Table 1.** Comparison between a Normal Ball and the Ball with a Reflective Sheet.

Note: Ten balls were compared for each type of ball using a stimp meter.

**Supplementary Table 2.** Sorting of Individual Results for CE MLD of Final Ball Position.


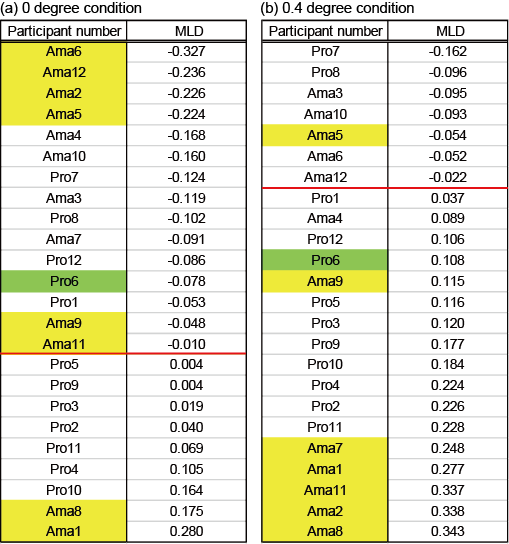


Note: CE: constant error, MLD: mediolateral direction. The amateurs, classified into (d) and (e) in Figure 3, are shown in yellow in this table. One professional classified in Figure 3(d) is shown in green. Positive MLD values indicate that the final ball position (FBP) was to the right of the target’s center, and negative values indicate that the FBP was to the left of the target’s center.

**
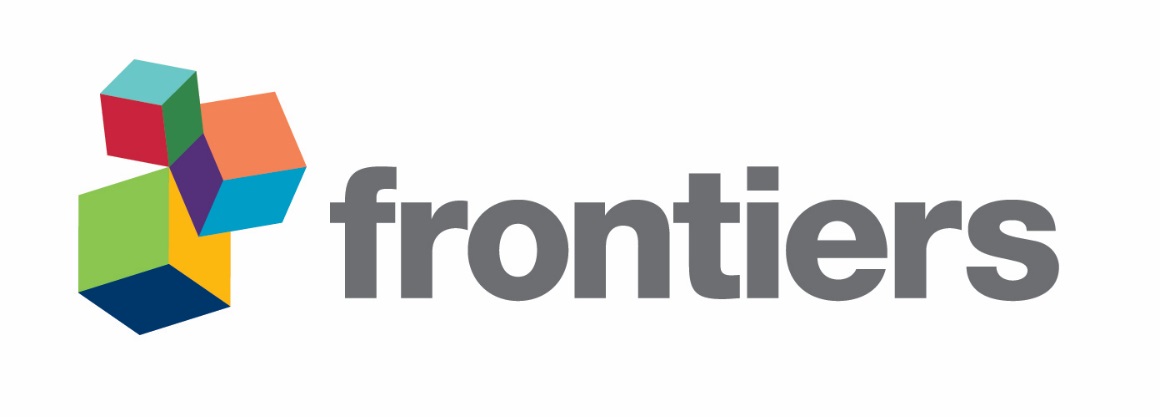
**
